# Blend Electrospinning of *Nigella sativa*-Incorporating PCL/PLA/HA Fibers and Its Investigation for Bone Healing Applications

**DOI:** 10.1021/acsomega.3c07523

**Published:** 2024-02-19

**Authors:** Mohammad Moghaddasi, Muhammed Mustafa Mert Özdemir, Ali Torabkhani Noshahr, Hüseyin Murat Özadenç, Busra Oktay, Ayşe Betül Bingöl, Pelin Pelit Arayıcı, Azime Eraslan, İlkay Şenel, Mariana Carmen Chifiriuc, Cem Bülent Üstündağ

**Affiliations:** † Department of Bioengineering, Faculty of Chemical and Metallurgical Engineering, Yıldız Technical University, 34220 Istanbul, Türkiye; ‡ Health Biotechnology Joint Research and Application Center of Excellence, Esenler, 34220 Istanbul, Türkiye; § Central Research Laboratory, Yıldız Technical University, Esenler, 34220 Istanbul, Türkiye; ∥ Department of Microbiology and Immunology, Faculty of Biology, University of Bucharest, 060101 Bucharest, Romania; ⊥ Research Institute of the University of Bucharest (ICUB), 050568 Bucharest, Romania; # Romanian Academy, 050045 Bucharest, Romania

## Abstract

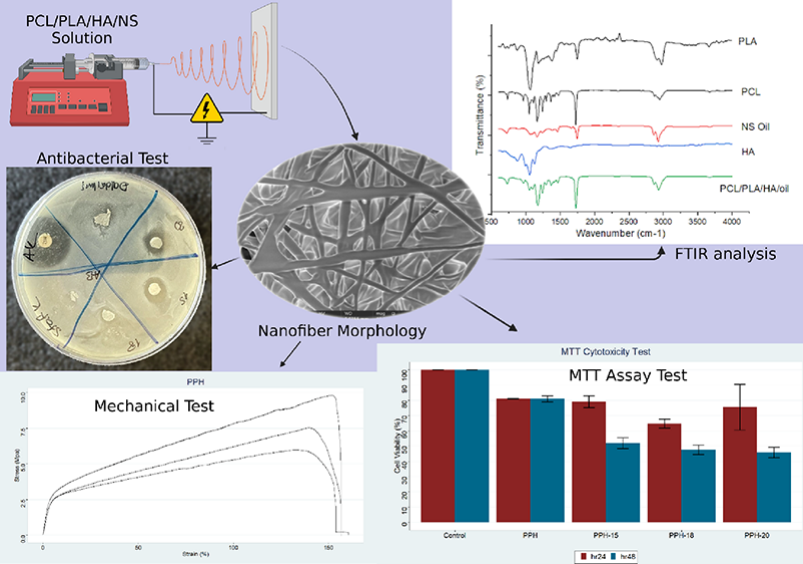

One of the well-known postoperative complications that requires a number of prophylactic and curative treatments is infection. The implications of postsurgical infections are further exacerbated by the emergence of antibiotic-resistant strains. Reduced effectiveness of synthetic antibiotics has led to an interest in plant-based substances. Extracts obtained from *Nigella sativa* have been shown to possess effective anti-infectious agents against bacteria frequently seen in bone infections. In this study, a fiber-based bone scaffold containing polycaprolactone, poly(lactic acid), and hydroxyapatite with *N. sativa* oil at varying concentrations was developed. Solvent electrospinning was used to fabricate the fibers with the specified composition. According to FE-SEM analysis, fibers with average diameters of 751 ± 82, 1000 ± 100, 1020 ± 90, and 1223 ± 112 nm were formed and successful integration of *N. sativa* oil into the fiber’s structure was confirmed via FTIR. *Staphylococcus aureus* showed moderate susceptibility against the fibers with a maximum inhibition zone diameter of 11.5 ± 1.6 mm. MTT assay analysis exhibited concentration-dependent cell toxicity against fibroblast cells. In short, the antibacterial fibers synthesized in this study possessed antibacterial properties while also allowing moderate accommodation of CDD fibroblast cells at low oil concentrations, which can be a potential application for bone healing and mitigating postsurgical infections.

## 1 Introduction

Bone is a dense and hierarchical connective tissue that plays a crucial role in movement, protection, mineral homeostasis, and endocrine regulation. The primary constituents of this tissue are the osseous cells, a composite extracellular matrix containing organic and inorganic molecules, and water.^1^ As opposed to the soft tissues, bone has the innate ability to regenerate to the original state without forming fibrotic scars in the injured area, thus recovering its initial mechanical and functional properties.^2^ In the case of serious medical conditions such as trauma, tumors, congenital, and infections, however, the size of the defects exceeds the critical limit, forming bone lesions. As a consequence of the significant gap created, this tissue is unable to regenerate independently and requires medical intervention.^3^ Bone transplantation, particularly autogenous bone grafting, is extensively utilized to treat bone lesions due to its good osseointegration and osteoconductivity capabilities. Nevertheless, allograft and autograft techniques used in bone transplantation accompany certain serious disadvantages. Donor site morbidity, infection, limited source, and immune rejection are especially of paramount importance and need to be considered while planning for the aforementioned procedures.^4^

To overcome the disadvantages of current treatment options and provide patients with a safer alternative, bone tissue engineering (BTE) principles have been widely utilized by researchers in the field. Analogous to the other areas of tissue engineering, BTE employs a combination of scaffolds, cells, and bifunctional molecules as a supporting platform to facilitate regeneration at the site of the bone injury.^5^ Among the fundamental elements involved in BTE, the fabrication of suitable scaffolds with desired characteristics such as biodegradability, biocompatibility, and adequate mechanical strength has been a matter of significant importance.^6^ The main purpose of scaffolds is to mimic the structure and function of the natural bone extracellular matrix (ECM), which can provide a three-dimensional (3D) environment with the physical properties necessary for bone repair, promoting adhesion, proliferation, and differentiation.^7,8^ The fundamental building blocks of scaffolds, known as biomaterials, have been integral to the favorable results of BTE. An optimal biomaterial should exhibit *in vivo* noncytotoxicity, biocompatibility, biodegradability, bioactivity, and osteoconductivity.^9,10^ Given the diverse requirements of scaffolds, composite materials, which combine two or more materials with distinct properties, have found extensive application in the field of bone tissue engineering.^11^

Polylactic acid (PLA) and polycaprolactone (PCL) are among the synthetic polymers widely used in BTE. PLA and PCL are preferred due to their biocompatibility, biodegradation, nontoxic effects, and controllable degradation rates after administration to the human body.^12^ PCL is a semicrystalline polyester widely used as a biomaterial in medical applications. PCL has a low melting point (55 °C) and favorable properties for bone tissue regeneration such as porosity and reabsorption.^10^ PLA is a biodegradable synthetic polymer, and its compressive strength (2–39 MPa) is similar to that of natural bone (2–12 MPa).^13,14^ Although PLA has excellent mechanical properties, this polymer is inherently more brittle, shows less flexibility, and can degrade quickly compared to PCL.^15^ Furthermore, pure PCL has low surface energy, resulting in a lack of binding signals and consequent inhibition of cell adhesion and proliferation on the surface.^16^ Therefore, blending the aforestated polymers can be an effective way to develop a new biomaterial to overcome the limitations of each polymer, thus enhancing the material’s properties. In addition, hydroxyapatite is a bioceramic with chemical and structural similarities to the mineral phase of bone ECM.^17^ The incorporation of a bioactive agent such as HA into the material’s structure leads to improvement in scaffold properties that can greatly affect biocompatibility, mechanical strength, and hydrophilicity.^16^ Due to the similar size of apatite in natural bones, HA nanoparticles in the nanorange increase the differentiation and proliferation of bone cells and cause improved mineral deposition.^17,18^ It contains calcium, which can lead to an enhanced formation of new bone tissue.^10^

The occurrence of infection at the site of bone transplantation, which is further exacerbated by the emergence of antibiotic-resistant strains, necessitates BTE scaffolds to exhibit antibacterial properties to disrupt the function of infectious agents via different mechanisms of action. With the declining efficacy of synthetic antibiotics against antibiotic-resistant bacteria, there has been a shift toward plant-based products as possible alternatives. Plant oils and extracts, in particular, have been heavily investigated due to their various antibacterial content including terpenes and phenylpropanoids.^19^*Nigella sativa*, also known as black cumin, is a flowering plant that belongs to the Ranunculaceae family and is native to southwestern Asia.^20^ Essential oils derived from the seed of this plant contain various potent antibacterial phytochemicals, including thymoquinone, thymol, carvacrol, and *p*-cymene. Several studies have shown the effectiveness of *N. sativa* seed oil against Gram-negative (*Escherichia coli* and *Pseudomonas aeruginosa*) and Gram-positive strains (*Staphylococcus aureus* and *Bacillus subtilis*), the latter being more susceptible.^21,22^ Ali et al. investigated the antibacterial and wound healing efficiency of an electrospun PVA-NS nanofibrous mat. In this study, two distinct samples with 47 and 67% (w/v) oil ratios were prepared. *S. aureus* and *E. coli* were found to be highly susceptible and mildly susceptible in both samples, respectively.^23^ Moreover, Sharifi et al. showed increased cell viability and antibacterial properties of PCL/PLA/NS nanofibers made with double-nozzle electrospinning.^24^ Similar findings were reported by Kahdim et al., who made composite nanofibers of PCL/Chitosan/NS oil.^25^

In this study, to produce a biocompatible fibrous mat with antibacterial properties, NS extract at varying concentrations was combined with PLA, PCL, and HA. Subsequently, the obtained solutions were converted into fibers using blend solvent electrospinning. Field emission scanning electron microscopy (FE-SEM) was used to investigate the surface morphology. Fourier transform infrared spectroscopy (FTIR) was carried out to verify the presence of the desired functional groups. A disk diffusion test aided in the semiquantitative evaluation of the scaffolds’ antibacterial properties. Mechanical tests were performed to gain insight into the durability and strength of the fibers. Finally, the cytotoxicity of the samples was evaluated by a standard MTT assay test.

## 2 Experimental Part

### 2.1 Materials

Poly(lactic acid) (PLA; *M*_w_ = 6 kDa) and poly(ϵ-caprolactone) (PCL; *M*_w_ = 80 kDa) were purchased from Sigma-Aldrich. Hydroxyapatite (HA) was synthesized in the laboratory using a standard wet chemical method. Solvents *N*,*N*-dimethylformamide (DMF) and dichloromethane (DCM) were purchased from ISOLAB Chemicals. The SY002-2 Cold Pressed Black Cumin Oil was purchased from OnkaFarma (Turkey, Izmir).

### 2.2 Method

#### 2.2.1 Synthesis of Nanohydroxyapatite (n-HA)

As mentioned by Liu et al, the stoichiometric Ca/P ratio required to produce nanoscale HA is 1.67.^26^ Accordingly, calcium nitrate tetrahydrate (Ca(NO_3_)_2_·4H_2_O) was mixed with distilled water at 300–400 rpm. According to the stoichiometric Ca/P ratio, the proper amount of diammonium dihydrogen phosphate ((NH_4_)_2_HPO) was mixed separately in distilled water. Next, ammonium dihydrogen phosphate solution was added dropwise to the calcium nitrate tetrahydrate solution. The pH of the resulting solution was checked daily to keep it above 10 during incubation. The incubation period allowed the formation and maturation of nano-HA particles. The required pH value was reached by adding ammonia dropwise to the solutions at a pH value below 10. At the end of the incubation period, the mixture solution was mixed for 1 min, poured into centrifuge tubes evenly, and centrifuged at 3000 rpm for 5 min. After centrifugation, the supernatant was discarded. Next, the tubes were filled with pure water, shaken vigorously, and centrifuged again at 3000 rpm. Finally, the pellet in the tubes was removed with a spatula and transferred to a glass plate. Nanoparticles were obtained by drying HA in an oven at 100–120 °C overnight.

#### 2.2.2 Solution Preparation

PCL/PLA/HA solution (4:1:0.25 mass ratio) with an overall concentration of 10 wt % was prepared by dissolving each component in a mixture solvent of DCM/DMF (60/40). Subsequently, they were left to be stirred for 24 h under ambient conditions. After that, the solution was sonicated until a homogeneous mixture was obtained to dissolve any polymer residues remaining in the solution. Next, NS oil at varying concentrations of 15, 18, and 20 wt % (labeled as PPH-15, PPH-18, and PPH-20, respectively) was added to the same solution in separate beakers along with the control solution (PPH) and thoroughly mixed for another 24 h.

#### 2.2.3 Electrospinning

For the electrospinning process, the PCL/PLA/HA/NS oil solution samples were loaded into a 20 mL plastic syringe with a metal needle (21 gauge, blunt end). The setup consisted of a voltage power supply (7000 Series Power Supply by Genvolt), a digital syringe pump (New Era pump systems), and a metal collector plate wrapped with aluminum foil. Electrospinning parameters were varied in a certain range according to the different oil concentrations to find the optimized conditions. The distance between the syringe tip and the collector plate was adjusted to 10–15 cm. The flow rate was set at a range between 1.2 and 2.5 mL/h depending on the oil concentration. Voltage was varied between 15 and 17 kV to obtain a steady fiber jet. The optimized electrospinning parameters were determined as 15 kV voltage, 2.3 mL/h flow rate, and 10 cm distance. After fibers were obtained under ambient conditions and relatively dry humidity, they were placed in a fume hood for the residual solvents to evaporate.

### 2.3 Characterization

#### 2.3.1 Morphological Analysis

The synthesized fibers with varying oil concentrations were observed under FE-SEM (Thermo Scientific Apreo 2S, Waltham, MA, USA). Prior to imaging, the scaffolds were coated with gold using a sputter coater and then scanned under 1 kV acceleration voltage and ×10000 g magnification scale. Based on FE-SEM images at a magnification of 1000× g, fiber diameters were determined using image analysis software.

#### 2.3.2 Chemical Analysis

By using FTIR, the chemical compositions of the produced PCL/PLA/HA and PCL/PLA/HA/oil fiber scaffolds were examined. Each sample was combined with KBr before being converted to disks. A Fourier transform infrared spectrometer (Equinox 55 LS 101, Bruker, Germany) was used to record the samples’ infrared (IR) spectra in the 400–4000 cm^–1^ wavelength range.

#### 2.3.3 Mechanical Test

Mechanical testing of PPH, PPH-15, PPH-18, and PPH-20 fiber scaffolds was performed in triplicate using a Shimadzu tensile machine (EZ-X, 346-57300-44, Kyoto, Japan) at a load capacity of 10 N and an extension speed of 5 mm/min. Square strips of fibers with different oil concentrations were cut, and their respective tensile and elastic moduli were estimated from the obtained stress–strain curves.

#### 2.3.4 Antibacterial Test

Antibacterial properties of PPH, PPH-15, PPH-18, and PPH-20 fibers were investigated against *S. aureus* (Gram-positive) and *E. coli* (Gram-negative) bacteria using a disk diffusion technique. The samples were all formed into disks of nearly the same size. For 24 h, *S. aureus* and *E. coli* bacteria were cultured at 37 ± 0.1 °C. Next, 0.01 mL of the aforementioned culture media was injected into sterilized Petri dishes. Each infected Petri dish received 15 mL of Muller–Hinton agar (Merck). By lightly pressing, fibrous disks were placed on the solid agar medium. The treated Petri dishes were incubated at 37 ± 0.1 °C for 16–24 h. The inhibitory zones that developed on the medium were eventually quantified. For each test strain, antibacterial activity experiments were conducted thrice, and average measurements were computed.

#### 2.3.5 MTT Assay

3-(4,5-Dimethyl-thiazol-2yl)-2,5-diphenylterazolium bromide (MTT) test was carried out to investigate the *in vitro* cytotoxicity of the fibrous scaffolds. CCD-1072-SK human fibroblast cells were seeded at a density of 105 cells/mL and incubated overnight in an incubator at 37 °C, containing 5% CO_2_ and a humid environment. Identical size fiber samples were prepared and sterilized with UV for 2 h. Subsequently, fiber samples were placed in 96-well plates containing 200 μL of DMEM culture medium along with CDD fibroblast cells and incubated at 37 °C for 24 and 48 h separately. At the end of the incubation, MTT solution (5 mg/mL) was added after removing the DMEM medium of each well and left for incubation for 4 h. Finally, 200 μL of DMSO was added to emptied wells to solubilize formazan crystals. The absorbance of all of the wells was read at 570 nm in an ELISA plate reader. All of the culturing experiments were repeated in triplicate. Cell viability was investigated and quantified by comparing the absorbance to that of negative control at the 24 and 48 h mark.

## 3 Results and Discussion

### 3.1 Morphological Analysis of Nanofibers

**Figure 1:**
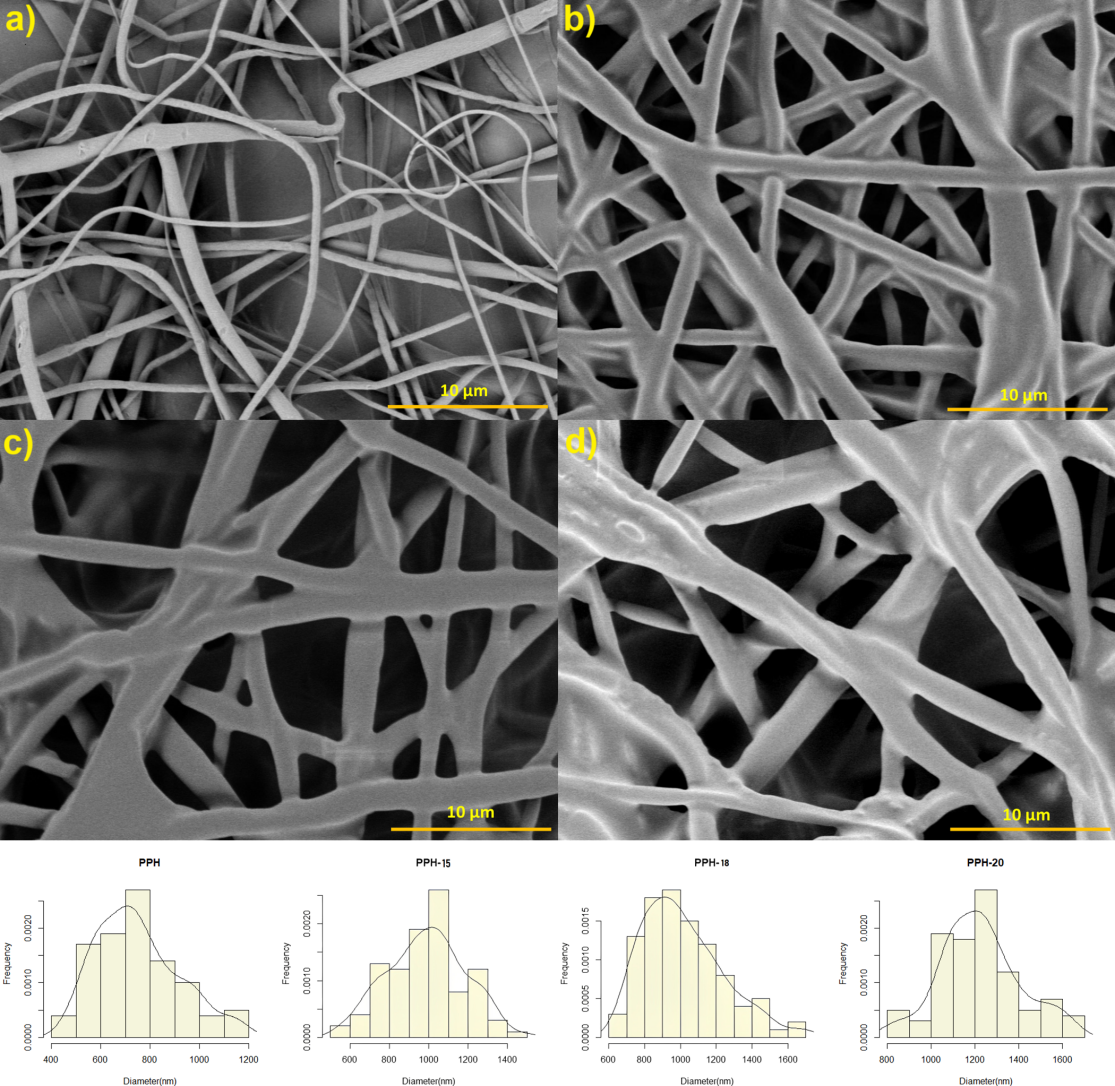
FE-SEM images of PPH (a), PPH-15 (b), PPH-18 (c), and PPH-20 (d) samples.

Morphology analysis of the PPH, PPH-15, PPH-18, and PPH-20 fibers was conducted using field emission scanning electron microscopy (FE-SEM). It was observed that the fibers from different samples were mostly bead-free and were evenly distributed with varying diameters (Figure 1). The formation of beads in certain areas, however, could be attributed to the slight heterogeneity that was caused by the addition of the NS extract, which resulted in different jet formation times. The mean average diameter of the PPH sample was found to be 751 ± 82 nm. Samples containing the NS extract showed a similar thick and bulky arrangement of the fibers. There was an increase in the diameter of PPH-15 (1000 ± 100 nm), PPH-18 (1020 ± 90 nm), and PPH-20 (1223 ± 112 nm) samples. Prior to electrospinning, with exceeding 20% NS concentration in the solution, phase separation of the components and the formation of polymer sediment were observed. This indicated that polymer solubility reached the saturation point in concentrations above 20%. Hence, the solubility of the polymers decreased proportional to the NS content and possibly led to the accumulation of polymers in certain areas, contributing to the increase in fibers’ diameters.^24^ Finally, there are areas where fibers have gathered and formed clumps, as shown in Figure 1d. This may be explained due to fibers ejecting in an unsteady state fashion because of the reduced polymer solubility with rising NS concentration.

### 3.2 Chemical Analysis of Nanofibers

**Figure 2:**
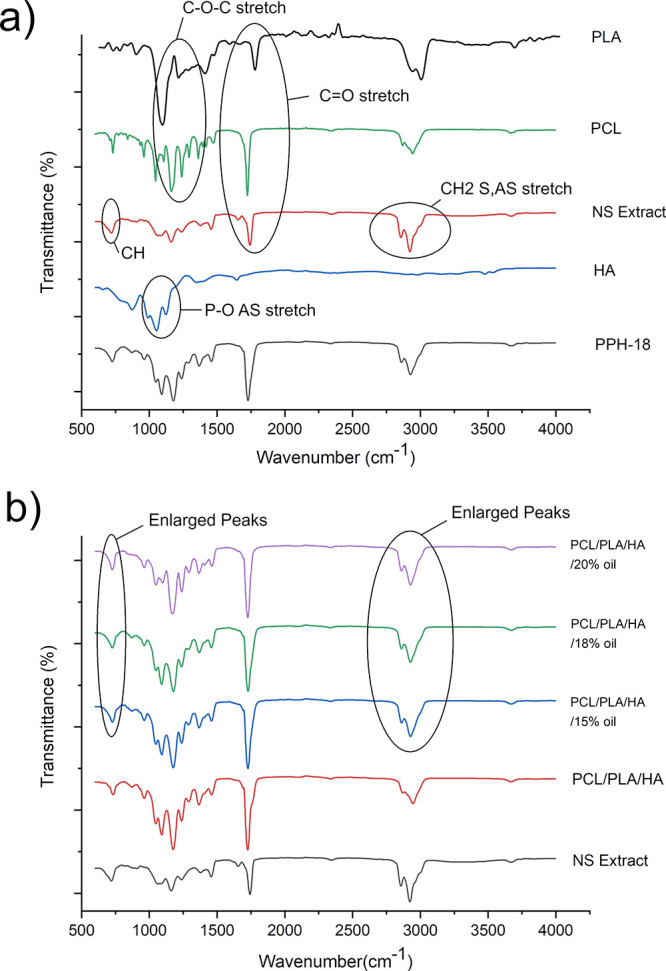
(a) FTIR spectra for PLA, PCL, NS oil, HA, and PPH-18 fibers. (b) FTIR spectra for NS oil, PPH, PPH-15, PPH-18, and PPH-20 fibers.

To investigate the chemical composition of the fibers and the functional groups present in the samples, we carried out FTIR analysis. Figure 2a displays the obtained transmittance graphs for PLA, PCL, NS oil, n-HA, and the PPH-18 sample. The FTIR spectrum of PCL and PLA at 1170–1250 cm^–1^ pertained to the symmetric and asymmetric C–O–C stretching vibration, 1290–1350 cm^–1^ to the C–O and C–C stretching vibration, and 1720–1760 cm^–1^ to the C=O stretching vibration, and ones at ∼2850 and 2950 cm^–1^ belonged to the symmetric and asymmetric CH_2_ stretching vibration.^27^ The stretching vibration of the O–H bond in the structure of hydroxyapatite was linked to the peaks at ∼ 650 cm^–1^. Furthermore, the P–O of the PO4^3–^ group exhibited an asymmetric stretching vibration, which is related to the primary peak of the phosphate group, which appeared in the range between 1000 and 1200 cm^–1^.^28^ As for the NS spectrum, peaks at 720 cm^–1^ (CH bending), 1180 cm^–1^ (CO stretch), 1463 cm^–1^ (CH bending), ∼1750 cm^–1^ (C=O stretching), and 2850–3100 cm^–1^ (CH_2_ symmetric and asymmetric stretching) were observed, which was consistent with findings of Rohman and Ariani.^29^Figure 2b shows the transmittance for PPH, PPH-15, PPH-18, and PPH-20 samples. The spectra of PPH samples containing varying oil concentrations exhibited similar peaks to those seen in the PCL/PLA/HA sample, which contained no oil due to the presence of the same functional groups. However, the sharp characteristic peak of NS oil at 2800–3000 cm^–1^ (CH_2_ symmetric and asymmetric stretching) was more pronounced in oil-containing samples, thus confirming its successful integration into the fiber’s structure. Moreover, a sharper peak at 720 cm^–1^ (CH bending) seen only in the NS oil graph is also detected in the composite fiber’s structure.

### 3.3 Mechanical Properties of Nanofibers

**Figure 3:**
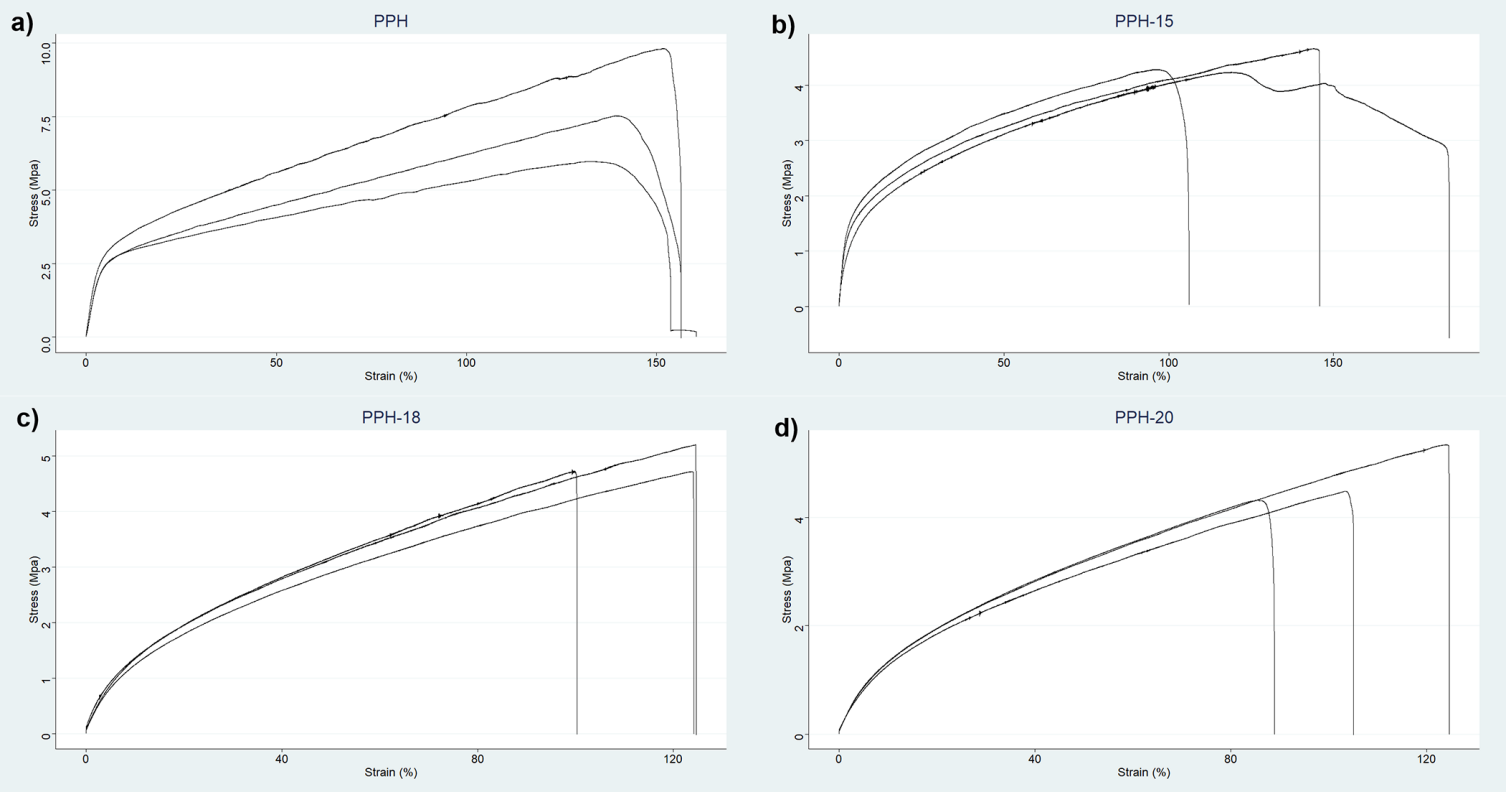
Stress–strain graphs of fibers: PPH (a), PPH-15 (b), PPH-18 (c), and PPH-20 (d).

**Figure 4:**
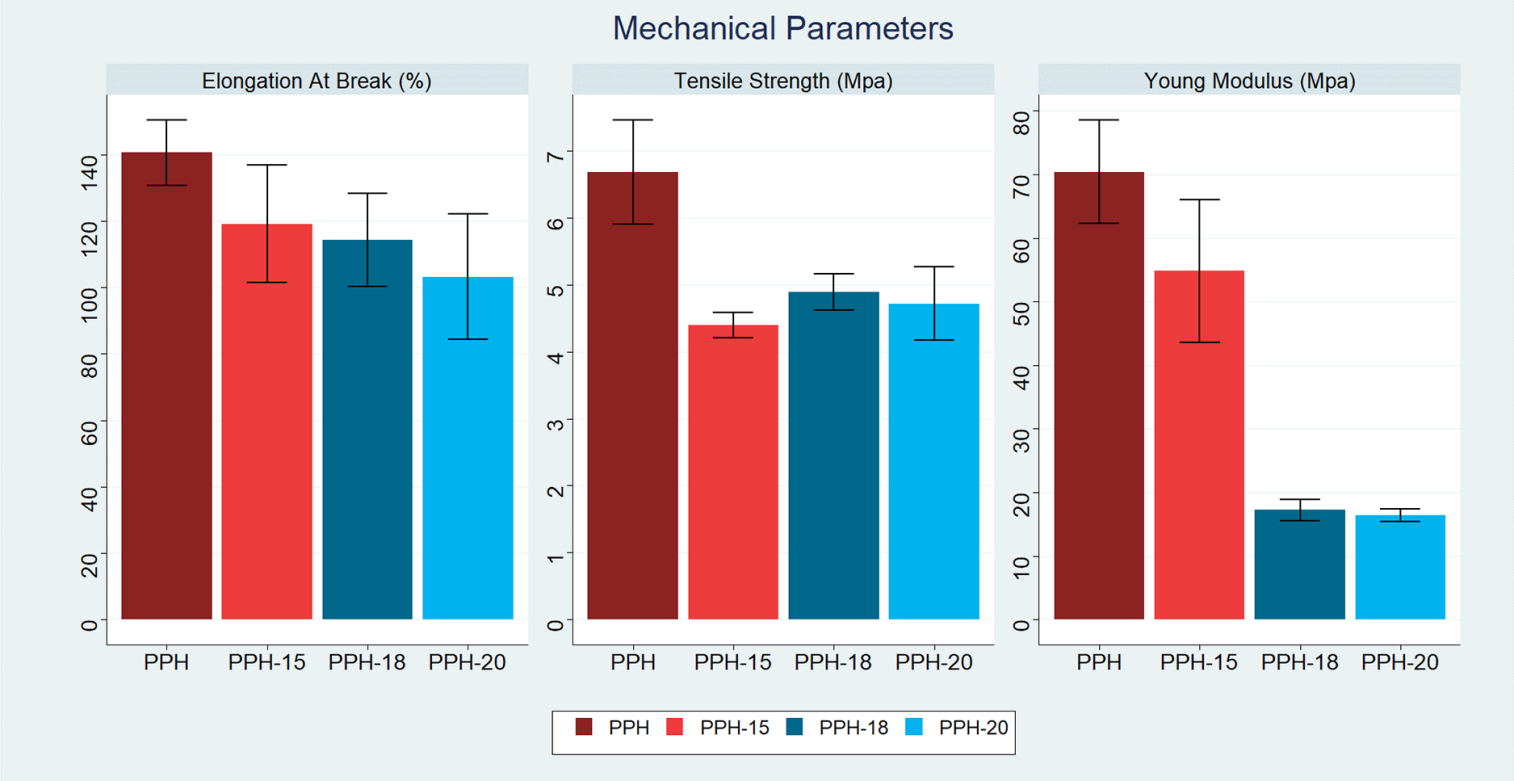
Mechanical parameters of PHH, PHH-15, PHH-18, and PHH-20 samples.

Tensile test results for the PPH, PPH-15, PPH-18, and PPH-20 fibers were converted into stress–strain graphs, as seen in Figure 3. Important mechanical properties including Young's modulus (YM), tensile strength (TS), and elongation at break (EB) were obtained using the aforementioned graphs, and they are summarized in Table 1. The incorporation of NS extract into the composite fibers’ structure affected their mechanical properties. TS was the highest for the fiber without oil (PPH, 6.69 ± 0.78 MPa) and had an average of 30% decrease at higher oil concentrations. This has been postulated to be due to the role of the NS extract as a plasticizing and regulatory agent of the polymer chains.^24^ Furthermore, fibers experienced an average of a 6% decrease in EB. A striking observation was the sudden decline of YM at PPH-18 and PPH-20 samples, exhibiting over a 70% decrease in value. As mentioned earlier, with increasing oil content, the decline of polymer solubility could have contributed to inhomogeneity in the fiber microstructure, thus creating stress points that led to a decrease in stiffness and, consequently, YM. Moreover, the addition of extracts such as NS at high concentrations can have a role in weakening the intermolecular interactions between polymer chains and cohesive forces within the material, making it less likely to resist deformation.^30,31^ Herbal extracts can be attributed to the disruption in the formation of crystalline regions that contribute to stiffness as well.^32,33^ In short, the incorporation of NS oil into the fiber’s structure exhibited a moderate decrease in TS and EB parameters, while a more significant decrease in YM was observed for the PPH-18 and PPH-20 samples, making the fibers with higher oil content less mechanically robust in comparison to PPH (Figure 4).

**Table 1:** Mechanical Properties of PHH, PHH-15, PHH-18, and PHH-20 Fibers

**sample**	**elastic modulus (MPa)**	**tensile strength (MPa)**	**elongation at break (%)**
PHH	70.43 ± 8.18	6.69 ± 0.78	140.65 ± 9.86
PHH-15	54.83 ± 11.22	4.40 ± 0.19	119.16 ± 17.67
PPH-18	17.23 ± 1.69	4.89 ± 0.27	114.27 ± 13.98
PPH-20	16.37 ± 0.99	4.72 ± 0.55	103.19 ± 19.91

### 3.4 Antibacterial Activity

**Figure 5:**
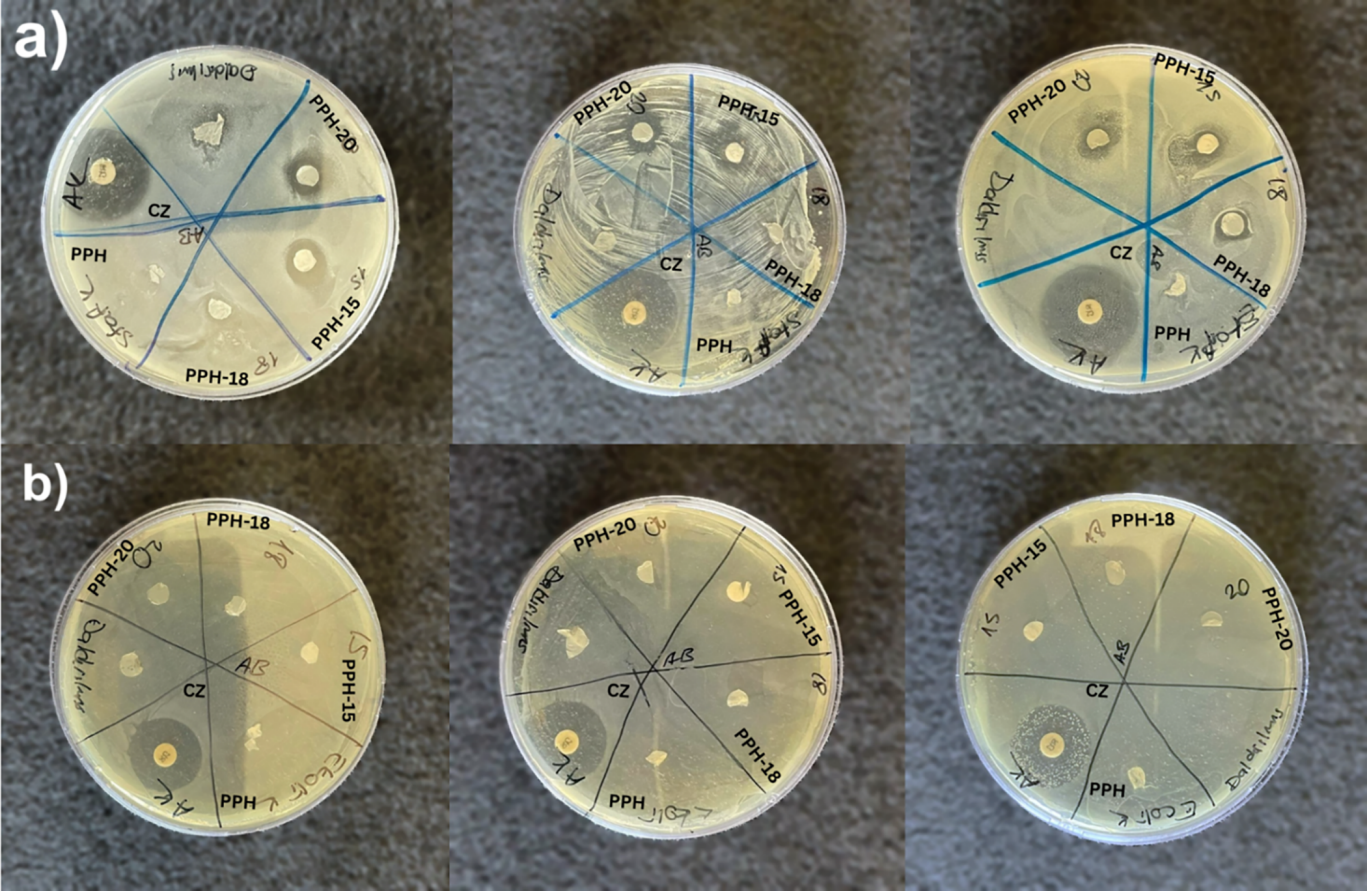
Disk diffusion test of PPH, PPH-15, PPH-18, and PPH-20 fibers against *S. aureus* (a) and *E. coli* (b).

**Figure 6:**
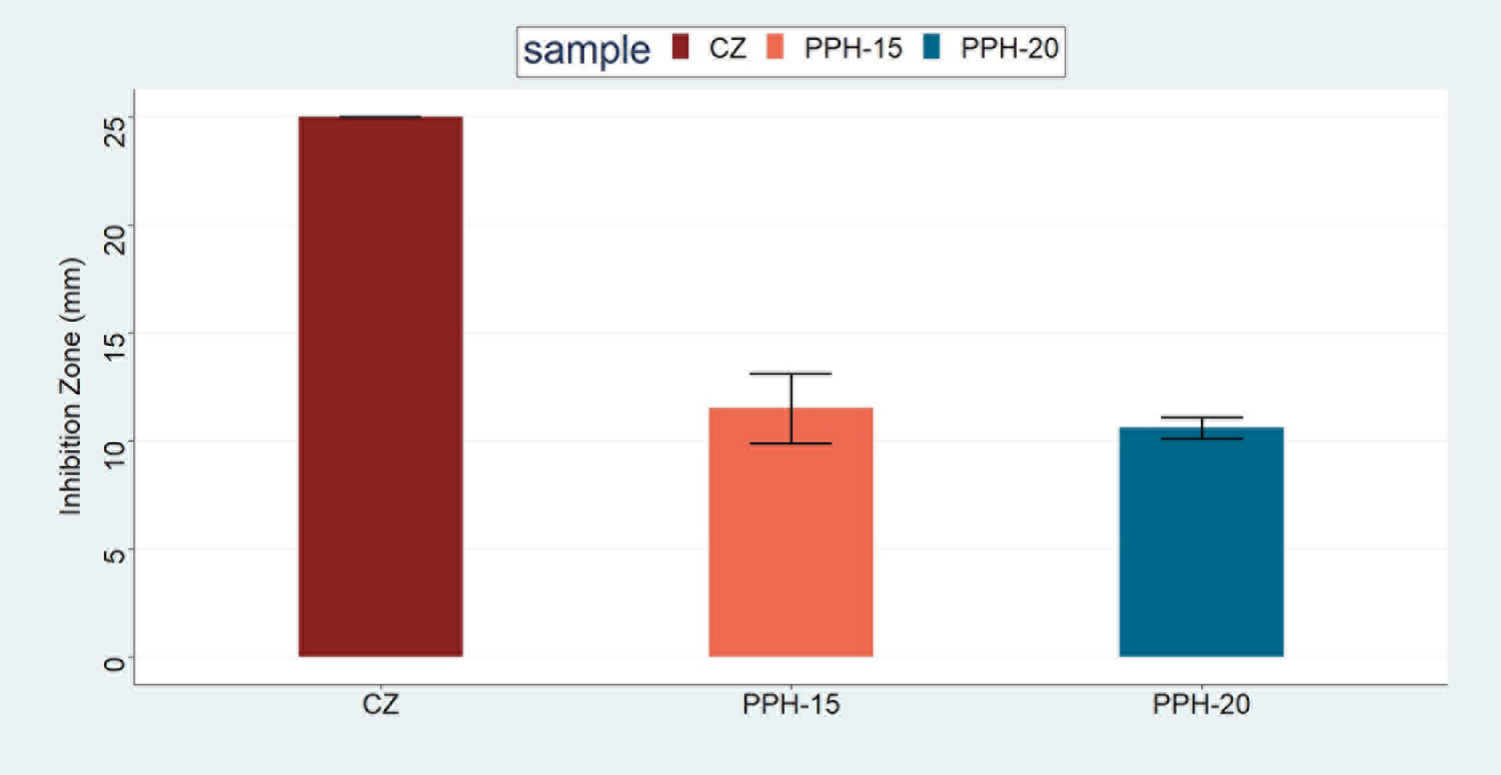
Mean diameter of inhibition zones formed around the PPH-15 and 20 samples against *S. aureus*.

By inhibiting biofilm formation, scaffolds with antibacterial properties play an important role in providing a functional environment for cell growth and tissue regeneration.^34^ In this study, the antibacterial potency of the prepared fibers with varying NS oil concentrations (PPH, PPH-15, PPH-18, and PPH-20) was tested against *S. aureus* and *E. coli* using a disk diffusion method (Figure 5a,b). After 24 h of incubation, the resulting inhibition zones around the disk-shaped fibers were measured and compared to that of cefazolin (CZ), which was used as a positive control (Table 2). Formation of inhibition zones against *S. aureus* was consistently observed around the PPH-15 (11.5 ± 1.6 mm) and PPH-20 (10.6 ± 0.5 mm) nanofibers (Figure 6). As for PPH-18, however, an inhibition zone of 9 mm was measured only in one repeat, while in others, *S. aureus* showed no susceptibility. This may be due to the unsterile sampling techniques prior to the antimicrobial test or failure in obtaining a homogeneous polymer/oil mixture in preliminary steps. On the other hand, *E. coli* exhibited no inhibition zone formation, making it resistant against the samples. Similar studies also found *E. coli* to be more resistant against NS oil-incorporating fibrous mats.^23,35,36^ This is due to the double-layered nature of Gram-negative strains that acts as a thicker barrier against antibacterial agents compared to that of Gram-positive bacteria.^26^ Constituents such as thymoquinone (TQ), thymohydroquinone (THQ), carvacrol, thymol, and terpenoids present in the NS oil increase the permeability of the cellular membrane, thus leading to its breakdown and release of cell content. TQ has also been reported to have antibiofilm formation properties that mitigate the oxidative activity of strains present in biofilms.^23,37^

**Table 2:** Diameter of Inhibition Zones Formed around Each Sample against *S. aureus*

**test**	**PPH**	**PPH-15**	**PPH-18**	**PPH-20**	**CZ**
#1	0 mm	12.5 mm	0 mm	10.8 mm	∼25 mm
#2	0 mm	9.6 mm	9 mm	11.0 mm	∼25 mm
#3	0 mm	12.4 mm	0 mm	10.0 mm	∼25 mm

### 3.5 MTT Assay

**Figure 7:**
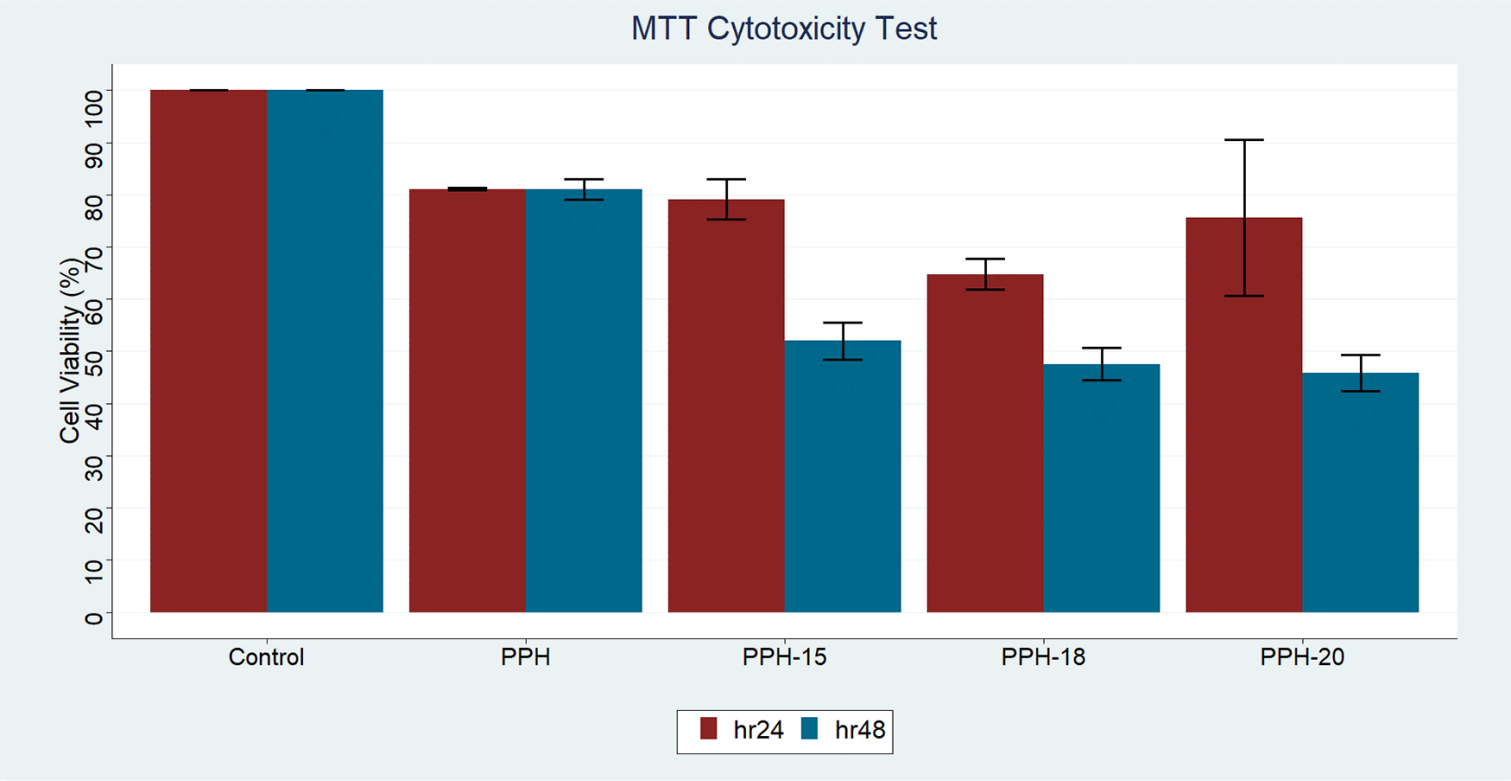
Cell viability for PPH, PPH-15, PPH-18, and PPH-20 fibers after 24 and 48 h of incubation.

The effect of PPH, PPH-15, PPH-18, and PPH-20 fiber scaffolds on the survival of CDD fibroblast cells for the 24th and 48th hours was evaluated using the MTT assay test. Figure 7 shows the cell viability of the scaffolds mentioned above at these time intervals. At the 24 h mark, the cell viability of the PPH-15 sample was comparable to that of the PPH sample, nearing 80%. This is in line with the findings of Sharifi et al., who demonstrated that NS-containing composite mats were noncytotoxic and enhanced hMSC cell migration and proliferation based on their MTT results.^24^ For the PPH-18 and PPH-20 samples at this time interval, however, lower cell viabilities of 65 and 75% were observed, respectively. This may have been an indication of cytotoxicity against the fibroblast cells at higher oil concentrations. This situation was far more pronounced in the observations made at the 48 h mark at which there was a significant decline in cell viability (PPH-15: 52%; PPH-18 and PPH20: ∼45%). Ugur et al. observed that *N. sativa* had no cytotoxic effect on fibroblast cells up to the concentration of 1 μg/mL.^38^ In this case, the high oil concentration utilized in the study has exceeded the aforementioned range and thus exhibited cytotoxic effects against the fibroblast cells. As a result, to provide a suitable environment for cell survival in the presence of herbal extracts like that of NS, we recommend that the utilization of excess extract is to be avoided and the investigation of optimal ratios for cell proliferation to be investigated at moderate concentrations.

## 4 Conclusions

In this study, PCL/PLA/HA/oil fibers were obtained by electrospinning. The NS extract was added to the PLA and PCL polymer solutions as an antibacterial agent. Subsequently, the morphological, mechanical, chemical, and cytotoxic characteristics of fibers were examined. Our findings demonstrated that boosting the concentration of the NS extract increased the fibers’ diameter. The results of the chemical analysis of the fibrous composite by FTIR spectroscopy validated the addition of *N. sativa* to the PPH samples. According to mechanical tests, loading fibers with the NS extract reduced the overall mechanical properties of the scaffolds. Moreover, the extract’s inclusion in fibers gave them an antibacterial effect. It was evident that NS-incorporating fibers exhibited antibacterial behavior against Gram-positive bacteria due to the formation of inhibitory zones surrounding the fiber samples. Finally, MTT findings demonstrated that with increasing NS extract concentration in the fibers, the cytotoxic effect against the fibroblast cells was more pronounced. Considering the results, biocompatible PCL/PLA/HA/NS scaffolds can be used as antibacterial agents in bone tissue engineering given that the dosage of NS is adjusted in an appropriate range.

## References

[ref1] Ishida Y.; Gao R.; Shah N.; Bhargava P.; Furune T.; Kaul S. C.; Terao K.; Wadhwa R. Anticancer activity in honeybee propolis: Functional insights to the role of caffeic acid phenethyl ester and its complex with γ-cyclodextrin. Integrative cancer therapies 2018, 17 (3), 867–873. 10.1177/1534735417753545. 29390900 PMC6142091

[ref2] Carr N. J. The pathology of healing and repair. Surgery (Oxford) 2022, 40 (1), 13–19. 10.1016/j.mpsur.2021.11.003.

[ref3] Manzini B. M.; Machado L. M. R.; Noritomi P. Y.; da Silva J. V. L. Advances in Bone tissue engineering: A fundamental review. J. Biosci. 2021, 46, 1–18. 10.1007/s12038-020-00122-6. 33737501

[ref4] Zhu G.; Zhang T.; Chen M.; Yao K.; Huang X.; Zhang B.; Li Y.; Liu J.; Wang Y.; Zhao Z. Bone physiological microenvironment and healing mechanism: Basis for future bone-tissue engineering scaffolds. Bioactive materials 2021, 6 (11), 4110–4140. 10.1016/j.bioactmat.2021.03.043. 33997497 PMC8091181

[ref5] Oktay B.; Özerol E. A.; Ustundag C. B. Graphene-based materials for bone tissue engineering. Sigma J. Eng. Nat. Sci 2022, 41 ( (3), ).

[ref6] Oktay A.; Yilmazer H.; Przekora A.; et al. Corrosion response and biocompatibility of graphene oxide (GO)serotonin (Ser) coatings on Ti6Al7Nb and Ti29Nb13Ta4.6Zr (TNTZ) alloys fabricated by electrophoretic deposition (EPD). *Materials Today*. Communications. 2023, 34, 105236 10.1016/j.mtcomm.2022.105236.

[ref7] Alonzo M.; Primo F. A.; Kumar S. A.; Mudloff J. A.; Dominguez E.; Fregoso G.; Ortiz N.; Weiss W. M.; Joddar B. Bone tissue engineering techniques, advances, and scaffolds for treatment of bone defects. Curr. Opin. Biomed. Eng. 2021, 17, 100248 10.1016/j.cobme.2020.100248. 33718692 PMC7948130

[ref8] Qu H.; Fu H.; Han Z.; Sun Y. Biomaterials for bone tissue engineering scaffolds: A review. RSC Adv. 2019, 9 (45), 26252–26262. 10.1039/C9RA05214C. 35531040 PMC9070423

[ref9] Arif Z. U.; Khalid M. Y.; Noroozi R.; Sadeghianmaryan A.; Jalalvand M.; Hossain M. Recent advances in 3D-printed polylactide and polycaprolactone-based biomaterials for tissue engineering applications. Int. J. Biol. Macromol. 2022. 10.1016/j.ijbiomac.2022.07.140 35896130

[ref10] Murugan S.; Parcha S. R. Fabrication techniques involved in developing the composite scaffolds PCL/HA nanoparticles for bone tissue engineering applications. J. Mater. Sci.: Mater. Med. 2021, 32 (8), 93 10.1007/s10856-021-06564-0. 34379204 PMC8357662

[ref11] Rajak D. K.; Pagar D. D.; Kumar R.; Pruncu C. I. Recent progress of reinforcement materials: A comprehensive overview of composite materials. Journal of Materials Research and Technology 2019, 8 (6), 6354–6374. 10.1016/j.jmrt.2019.09.068.

[ref12] Harmanci S.; Dutta A.; Cesur S.; Sahin A.; Gunduz O.; Kalaskar D. M.; Ustundag C. B. Production of 3D Printed Bi-Layer and Tri-Layer Sandwich Scaffolds with Polycaprolactone and Poly (vinyl alcohol)-Metformin towards Diabetic Wound Healing. Polymers 2022, 14 (23), 5306 10.3390/polym14235306. 36501700 PMC9736052

[ref13] Komal U. K.; Lila M. K.; Singh I. PLA/banana fiber based sustainable biocomposites: A manufacturing perspective. Composites Part B: Engineering 2020, 180, 107535 10.1016/j.compositesb.2019.107535.

[ref14] Oktay B.; Ahlatcıoğlu Özerol E.; Sahin A.; Gunduz O.; Ustundag C. B. Production and Characterization of PLA/HA/GO Nanocomposite Scaffold. ChemistrySelect 2022, 7 (30), e202200697 10.1002/slct.202200697.

[ref15] Wang W.; Zhang B.; Li M.; Li J.; Zhang C.; Han Y.; Wang L.; Wang K.; Zhou C.; Liu L. 3D printing of PLA/n-HA composite scaffolds with customized mechanical properties and biological functions for bone tissue engineering. Composites, Part B 2021, 224, 109192 10.1016/j.compositesb.2021.109192.

[ref16] Hassanajili S.; Karami-Pour A.; Oryan A.; Talaei-Khozani T. Preparation and characterization of PLA/PCL/HA composite scaffolds using indirect 3D printing for bone tissue engineering. Materials Science and Engineering: C 2019, 104, 109960 10.1016/j.msec.2019.109960. 31500051

[ref17] Shi H.; Zhou Z.; Li W.; Fan Y.; Li Z.; Wei J. Hydroxyapatite based materials for bone tissue engineering: A brief and comprehensive introduction. Crystals 2021, 11 (2), 149 10.3390/cryst11020149.

[ref18] Ozder M. N.; Çiftçi F.; Rencuzogullari O.; Arisan E. D.; Ustündag C. B. In situ synthesis and cell line studies of nano-hydroxyapatite/graphene oxide composite materials for bone support applications. Ceram. Int. 2023, 49 (9), 14791–14803. 10.1016/j.ceramint.2023.01.075.

[ref19] Yu Z.; Tang J.; Khare T.; Kumar V. The alarming antimicrobial resistance in ESKAPEE pathogens: Can essential oils come to the rescue?. Fitoterapia 2020, 140, 104433 10.1016/j.fitote.2019.104433. 31760066

[ref20] Mariod A.; Saeed Mirghani M.; Hussein I. Nigella sativa L. black cumin. Unconventional oilseeds and oil sources 2017, 73–80. 10.1016/B978-0-12-809435-8.00013-5.

[ref21] Bakathir H. A.; Abbas N. A. Detection of the antibacterial effect of nigella sativa ground seedswith water. Afr. J. Tradit., Complementary Altern. Med. 2011, 8 ( (2), ). 10.4314/ajtcam.v8i2.63203 PMC3252685 22238497

[ref22] Shafodino F. S.; Lusilao J. M.; Mwapagha L. M. Phytochemical characterization and antimicrobial activity of Nigella sativa seeds. PloS one 2022, 17 (8), e0272457 10.1371/journal.pone.0272457. 35926002 PMC9352024

[ref23] Ali A.; Mohebbullah M.; Shahid M. A.; Alam S.; Uddin M. N.; Miah M. S.; Jamal M. S. I.; Khan M. S. PVA-Nigella sativa nanofibrous mat: antibacterial efficacy and wound healing potentiality. Journal of the Textile Institute 2021, 112 (10), 1611–1621. 10.1080/00405000.2020.1831168.

[ref24] Sharifi M.; Bahrami S. H.; Nejad N. H.; Milan P. B. Electrospun PCL and PLA hybrid nanofibrous scaffolds containing Nigella sativa herbal extract for effective wound healing. J. Appl. Polym. Sci. 2020, 137 (46), 49528 10.1002/app.49528.

[ref25] Kahdim Q. S.; Abdelmoula N.; Al-Karagoly H.; Albukhaty S.; Al-Saaidi J. Fabrication of a polycaprolactone/Chitosan nanofibrous scaffold loaded with nigella sativa extract for biomedical applications. BioTech 2023, 12 (1), 19 10.3390/biotech12010019. 36810446 PMC9944449

[ref26] Liu H.; Yazici H.; Ergun C.; Webster T. J.; Bermek H. An in vitro evaluation of the Ca/P ratio for the cytocompatibility of nano-to-micron particulate calcium phosphates for bone regeneration. Acta biomaterialia 2008, 4 (5), 1472–1479. 10.1016/j.actbio.2008.02.025. 18394980

[ref27] Marei N. H.; El-Sherbiny I. M.; Lotfy A.; El-Badawy A.; El-Badri N. Mesenchymal stem cells growth and proliferation enhancement using PLA vs PCL based nanofibrous scaffolds. Int. J. Biol. Macromol. 2016, 93, 9–19. 10.1016/j.ijbiomac.2016.08.053. 27554939

[ref28] Asghari F.; Salehi R.; Agazadeh M.; Alizadeh E.; Adibkia K.; Samiei M.; Akbarzadeh A.; Aval N. A.; Davaran S. The odontogenic differentiation of human dental pulp stem cells on hydroxyapatite-coated biodegradable nanofibrous scaffolds. International Journal of Polymeric Materials and Polymeric Biomaterials 2016, 65 (14), 720–728. 10.1080/00914037.2016.1163564.

[ref29] Rohman A.; Ariani R. Authentication of Nigella sativa seed oil in binary and ternary mixtures with corn oil and soybean oil using FTIR spectroscopy coupled with partial least square. Sci. World J. 2013, 2013. 10.1155/2013/740142 PMC3844219 24319381

[ref30] 30Pedram Rad Z.; Mokhtari J.; Abbasi M. Preparation and characterization of Calendula officinalis-loaded PCL/gum arabic nanocomposite scaffolds for wound healing applications. Iran Polym. J. 2019, 28 (1), 51–63. 10.1007/s13726-018-0674-x.

[ref31] Syafiq R.; Sapuan S. M.; Zuhri M. Y. M.; et al. Antimicrobial Activities of Starch-Based Biopolymers and Biocomposites Incorporated with Plant Essential Oils: A Review. Polymers. 2020, 12 (10), 2403 10.3390/polym12102403. 33086533 PMC7603116

[ref32] Pourhojat F.; Sohrabi M.; Shariati S.; Mahdavi H.; Asadpour L. Evaluation of poly ε-caprolactone electrospun nanofibers loaded with Hypericum perforatum extract as a wound dressing. Res. Chem. Intermed. 2017, 43 (1), 297–320. 10.1007/s11164-016-2623-7.

[ref33] Radisavljevic A.; Stojanovic D. B.; Petrovic M.; Radojevic V.; Uskokovic P.; Rajilic-Stojanovic M. Electrospun polycaprolactone nanofibers functionalized with Achillea millefolium extract yield biomaterial with antibacterial, antioxidant and improved mechanical properties. J. Biomed. Mater. Res., Part A 2023, 111 (7), 962–974. 10.1002/jbm.a.37481. 36571468

[ref34] Feng B.; Wang S.; Hu D.; Fu W.; Wu J.; Hong H.; Domian I. J.; Li F.; Liu J. Bioresorbable electrospun gelatin/polycaprolactone nanofibrous membrane as a barrier to prevent cardiac postoperative adhesion. Acta Biomaterialia 2019, 83, 211–220. 10.1016/j.actbio.2018.10.022. 30352286

[ref35] Kokoska L.; Havlik J.; Valterova I.; Sovova H.; Sajfrtova M.; Jankovska I. Comparison of chemical composition and antibacterial activity of Nigella sativa seed essential oils obtained by different extraction methods. Journal of food protection 2008, 71 (12), 2475–2480. 10.4315/0362-028X-71.12.2475. 19244901

[ref36] Harzallah H. J.; Noumi E.; Bekir K.; Bakhrouf A.; Mahjoub T. Chemical composition, antibacterial and antifungal properties of Tunisian Nigella sativa fixed oil. Afr. J. Microbiol. Res. 2012, 6 (22), 4675–4679. 10.5897/AJMR11.1073.

[ref37] Pignatelli C.; Perotto G.; Nardini M.; Cancedda R.; Mastrogiacomo M.; Athanassiou A. Electrospun silk fibroin fibers for storage and controlled release of human platelet lysate. Acta biomaterialia 2018, 73, 365–376. 10.1016/j.actbio.2018.04.025. 29673841

[ref38] Ugur A. R.; Dagi H. T.; Ozturk B.; Tekin G.; Findik D. Assessment of In vitro Antibacterial Activity and Cytotoxicity Effect of Nigella sativa Oil. Pharmacogn Mag. 2016, 12 (Suppl 4), S471–S474. 27761077 10.4103/0973-1296.191459 PMC5068126

